# Rainforest conservation as a strategy of climate policy

**DOI:** 10.1007/s10202-011-0095-9

**Published:** 2011-06-28

**Authors:** Dieter Cansier

**Affiliations:** Tübingen, Germany

## Abstract

Tropical forest conservation in developing countries has repeatedly been highlighted as a new element in international climate policy. However, no clear ideas yet exist as to what shape such a conservation strategy might take. In the present paper, we would like to make some observations to this end. It is shown how projects in order to reduce CO_2_-emissions resulting from deforestation and degradation (REDD) can be integrated into a system of tradable emission rights in an industrialised country and which requirements ought to be fulfilled. Instruments are emission credits and emission allowances. Driving actors interested in emission rights through forest projects may be private investors or the rainforest state itself. The efficiency of the system depends on a great extent on a binding reference path for the tolerable emissions from deforestation, which has been agreed upon and adhered to by the rainforest country by means of a forest law aimed at limiting deforestation. Our considerations lead us to conclude that the national baseline approach with an appropriate contribution by the rainforest country coupled with a decentralised system with private investors seems the most viable option. Since additional burdens are imposed on the rainforest country to some extent, a compromise could consist of agreeing on a moderate deforestation path, which is harmonised with the benefits from the forest projects. Combining both programmes (offset credits and emission allowances) is particularly attractive because all participants, and especially the industrialised country, benefit from it. The industrialised country can expand its climate conservation programme without any additional costs to a certain degree.

## Abstract (de)

Die Erhaltung der tropischen Wälder ist wiederholt als ein neues Element der internationalen Klimaschutzpolitik hervorgehoben worden. Jedoch gibt es bislang keine genaueren Vorstellungen, wie ein Schutzkonzept aussehen könnte. Dazu werden hier Überlegungen angestellt. Untersucht wird, wie ein Industrieland in sein System handelbarer CO_2_-Emissionsrechte Projekte zum Schutz der Regenwälder einbeziehen kann und welche Anforderungen dafür gelten sollten. Als Instrumente kommen Emissionsgutschriften und reguläre Emissionszertifikate in Betracht. Investoren können Private oder das Regenwaldland selbst sein (dezentraler oder zentraler Ansatz). Wesentliche Bedingung für die Wirksamkeit des Systems ist, dass ein bindender nationaler Referenzpfad für den tropischen Waldbestand vereinbart und durch Reform der Waldnutzungsrechte umgesetzt wird. Die Überlegungen laufen darauf hinaus, dass das nationale baseline-Konzept mit angemessenem Eigenbeitrag des Regenwaldlandes und das dezentrale System mit privaten Investoren am besten geeignet sind. Weil dem Regenwaldland mit den eigenen klimapolitischen Anstrengungen zusätzliche Lasten aufgebürdet werden, könnte ein Kompromiss darin bestehen, für sie einen moderaten Abbaupfad zu vereinbaren, der mit den Vorteilen aus den zusätzlichen Waldprojekten abgestimmt ist. Eine Kombination der beiden Programme, Emissionsgutschriften und reguläre Emissionszertifikate, erscheint besonders attraktiv, weil alle Beteiligten davon Vorteile haben. Für das Industrieland bietet sich die Chance, seinen Klimaschutz in bestimmten Grenzen ohne Mehrkosten auszuweiten.

## Introduction

Tropical forest conservation in developing countries has repeatedly been highlighted as a new element in international climate policy. There were also high hopes for the UN Conference on Climate Change in Mexico last December. However, no clear ideas yet exist as to what shape such a conservation strategy might take. In the present paper, we would like to make some observations to this end. A variety of legislative initiatives on climate conservation from the 2009/10 US Congress will serve as our starting point. Even if the initiatives failed, they unanimously included rainforest conservation as an integral part of climate policy and, for the first time, defined concrete requirements for a conservation policy.^1^

In this paper, we will focus on national policy: an industrialised country (the United States) makes an arrangement with a rainforest country to promote projects in order to reduce emissions resulting from deforestation and degradation (REDD) and determines both a specific set of instruments and a number of conditions. The regulations are integrated into a system of tradable emission allowances (cap-and-trade), a policy approach which, as opposed to emission taxes and standards, is considered particularly suitable for climate policy. The measures are based on the ‘do ut des’ principle—payment for services—meaning they differ from subsidy regulations. These considerations can easily be applied to an international rainforest policy for the industrialised world.

Current deforestations are so excessive that the potential for reducing and eventually stopping them could drastically reduce CO_2_ emissions—and most likely all at comparatively low costs. Were it to seize the opportunity, the industrialised world could get much closer to reaching its goal of limiting global warming to 2°C by 2050. According to estimates taking into account recent years, about 13–15% of global CO_2_ emissions are due to deforestation, which is roughly equivalent to the shares of industry, traffic and farming, respectively.^2^

## The cap-and-trade approach

Greenhouse gases are intended to be limited to a fixed absolute amount by way of a restricted annual allocation of tradable emission allowances. Only those who own allowances, as emitters within the system, are permitted to release the respective amount of CO_2_ and other greenhouse gases. In the US, the introduction of a national cap-and-trade system, partly based on the auctioning off of the allowances (roughly 18%), was planned for 2012 onwards. The system would provide two ways of taking into account REDD activities:

### Offset credits

Companies participating in the system would be allowed to offset a portion of their CO_2_ emissions by investing in activities outside the defined system (e.g. carbon capture and sequestration). They would receive credits allowing them to undertake the same amount of emissions within the system area. The recognition of REDD projects in developing countries would be new and, since the expected costs of these projects would be comparatively low, the national economy could be relieved considerably. Offset credits could be purchased both by emitters and third parties. For emitters, they could replace preventive measures at home; other purchasers could sell them in emissions trading. Because prices for allowances are decreasing, all emitters in the system would benefit from the lower costs. In case, the additional domestic emissions are equal to the reduced rainforest emissions, a constant level of total global emissions and an appropriately defined national climate goal would be maintained. In return for the agreed omission of deforestation, the rainforest country would receive a share of the returns, which would not only cover the costs but also provide a surplus.

An additional climate effect could be achieved by defining the exchange rate of emission reductions in credits, not to 1:1, but, for instance, to 1.25:1 (cf. ACESA). This allows for the pursuit of more ambitious climate goals. To this end, the industrialised country must forgo a part of otherwise possible cost reductions, since REDD projects are slightly more expensive for investors.

### Emission allowances set aside for reduced deforestation

The complete amount of annual allocated emission allowances is not distributed to the emitters, as some is reserved for investors engaging in REDD activities. In the US model, individuals and organisations would only be eligible if they were not emitters, including international organisations. Because the purchasers would sell their allowances on the US market, the allowances would eventually return there, meaning that after an initial cut in the allocation, the total amount given to domestic emitters would remain constant. So, the realisation of the domestic emission limit is combined with an additional positive climate effect in the rainforest countries. In this approach, unlike in the first programme, the industrialised country would bear additional costs for rainforest conservation. The burden would be carried by both the emitters, who would now have to buy allowances which they previously received free of charge, and the government, which would lose revenue by forgoing part of the auctioned amount. The winners would be the investors and project partners in the rainforest country.

The intention was that together, both conservation programmes were to bring about a considerable expansion of US climate conservation beyond what could be achieved domestically. Without these regulations, by 2020, emissions were expected to be reduced to the approximate level of 1990. As for the ACESA, it was estimated that both programmes would each lower this goal by around 6% by 2020, reaching minus 12% as compared to 1990.^3^ The US industry would have been substantially relieved from abatement costs as the offset programme would have been dominant.

The offset credits and allowances for reduced deforestation ought to be fully fungible. They should not carry with them any burdens from their realisation. The owners should not be restricted in the use or sale of the authorised amount of emission rights. All costs associated with a forest project would have to be carried by the investor. As soon as emission rights were granted by the public administrator, they should be valid indefinitely, even if the forest project was to fail at a later point.

For an industrialised nation, this system runs the risk that more emission rights would be assigned than emission reductions are created through forest activities. This would have different consequences for the two instruments. For the offset regulation (1:1 exchange), fewer emissions would be avoided than originally planned. The domestic goal would be violated. With the emission allowances programme, on the other hand, nothing would change for the emitters as a group. The domestic emission limit would be adhered to. However, the supplementary climate goal would not be met.

## National deforestation emission baseline

Due to the global climate orientation, the reference point of the forest strategy is the complete forest carbon stock of a partner country. Projects are intended to produce an additional and permanent effect on the climate, as opposed to the development path of deforestation that would otherwise occur. The current (annual) emission reductions must be measurable and verifiable. To this end, the state of forests and their carbon contents must be constantly recorded. According to experts, it is currently possible to measure reductions in CO_2_ emissions from deforestation in developing countries with a ‘sufficient level of accuracy’ (by satellite and with statistical methods on the ground).

Projects refer to forest areas which still exist, but whose stock will be endangered in the future. The investor and the licensing authority do not know whether or not deforestation would occur in the absence of the project. The basis of the assessment is uncertain, and manipulation by the original owners remains possible. A secure reference point is required and is assumed by the national baseline for the annual CO_2_ emissions due to deforestation. This baseline determines the maximum permitted deforestation emissions per period agreed upon between countries. Central to this is the importance of preventing a country from setting an excessively high reference emission quantity in order to receive funding for forest areas, which would have been conserved in any case. Aiming for permanency also entails that the forest stocks conserved during a particular period do not raise the baseline of a later period.

Some proponents of the rainforest policy suggest taking the business as usual path of deforestation emissions as a reference point, using deforestation rates of the past 5–10 years adjusted by the current state of politics. In this case, the country would not have to change its politics much at all, and relatively vague, the approach offers opportunities for manipulations. The US proposition, therefore, would set into place a stricter standard. The permissible deforestation quantity would be continually lowered over time until zero emission was reached after 20 years. This annual decline of the emissions would induce both a high binding effect with regard to the conservation strategy as well as involving the rainforest country in global climate policy. It would require forest rights be reformed. Illegal forest clearings would have to be stopped and legal deforestations limited. Instruments to accomplish this could include defining conservation areas, clarifying and restricting individual deforestation rights, more effective controls of, and penalties against, breaking the law, limiting government funding of logging as well as creating a sustainable infrastructure and settlement policy.

In both approaches, it must be ensured that the additionality and permanency of forest conservation not be undermined by counteracting reactions. So-called leakage must be prevented. This refers to the risk that the previous owners might shift their deforestation activities to other areas, that the current project owners might neglect their forest areas and that the state itself might conduct or at least tolerate forest degradations elsewhere. The very fact that this is possible is due to shortcomings in existing forest law, because previous users of the rainforest who relocate to other areas must be legally authorised to do so, and indeed are able to do so due to a lack of controls and sanctions. This becomes impossible if other people and organisations own the rights to use the land in question and if their rights are effectively protected. The same is true for leakage through the investor. Once his project is authorised, he might be tempted to neglect the necessary upkeep and protection of the land against exterior impacts, as this would result in cost savings for him. After all, his commitment would be considered ‘permanent’. It is also conceivable that, at a later date, an investor could intentionally carry out deforestations so as to acquire further revenue in addition to the gains from the emission rights. In such cases, forest law could also offer protection: once the authorised right of use is specified for the conservation of a forest area, its destruction would be illegal and forbidden.

The baseline quantities for each year should be constantly updated. It would be completely unrealistic to believe that a final path valid ‘for all time’ could be determined. The planning technique of sliding planning seems an obvious choice. In this approach, the overall planning period is predefined (i.e. 10–15 years). After each completed reference year of planning, a new year is added on to the end. The meanwhile improved level of knowledge allows for updating all planning data.

## Allocation of emission credits and emission allowances

The rainforest area is expected to receive emission rights to the same extent that actual emissions due to deforestation fall below the baseline. The macro-difference is seen as an indicator of the conservation projects authorised during the respective period. The difference must exceed a certain size in order to be measurable in the course of recording forest carbon stocks. Therefore, only bigger forest projects would be eligible for this type of policy (which would also make leakage more difficult).

The question is how to proceed if the macro-balance shows lower emission reductions than the sum of the individual project effects. In this case, other influences would have increased the actual emissions. What springs to mind are a lack of political enforcement, inaccurate measurements and natural impacts (droughts, fires, storms, flooding and tree diseases). One could adjust the macro-balance for these causes and set emissions lower than their actual quantity. In case of a lack of enforcement, however, this appears not to be the right way because too many emission rights would be distributed. The individual effects ought to be discounted. This type of discounting may seem unfair, as it means that the investors have to carry the burden of the government’s lack of enforcement; however, this consequence is an integral part of the system. In the case of forest degradation through natural forces, a new baseline should be chosen, since these effects are not related to the conservation policy. The damage would have occurred in any case and would certainly have caused the baseline to be raised by the partner states if the events could have been foreseen.

Time is another important aspect regarding the allocation of emission rights. The macro-balance can only be determined at the end of a one-year period, as forest carbon stocks are compared at the beginning and end of the period. Forest projects are, therefore, only authorised after, and not during, a period. This has important consequences: (1) It takes some time for investors to plan a project, and since there is no knowing beforehand to what extent their project will be authorised, they are faced with a particular time-related planning risk. They do not know if and to what extent discounting will occur. (2) Counteracting emission effects will occur later, after an activity has been authorised. Therefore, the authorisation of current projects is dependent upon the extent to which leakage was caused by prior projects. (3) The industrialised country will consider the macro-balance of a completed year and not the possible future effects when deciding about individual projects. There is no control mechanism for holding responsible a project operator who allows his forest area to degrade later. Liability for default is not possible.

The simple alternative to a national approach would be the introduction of a project-related baseline. This way, one would make do with a claim of additionality in the vicinity of forest activities. Any potential lack of enforcement or leakage outside of the area in question would not be recorded. The rainforest country would not be obliged to take legal action.^4^

## Private investors as actors

Driving actors are private investors interested in forest projects in order to gain financial benefits. They must purchase the user right of forest areas, which would otherwise be destroyed from those who currently are entitled (nations, states, local communities, individuals and private firms).^5^ Since this relates to the prevention of CO_2_ emissions, the user rights refer to the preservation of a forest stock rather than to other uses, such as the utilisation of the gene potential. The forest stocks must be preserved ‘indefinitely’. For investors, engaging in a REDD activity this means a long-term commitment, which can last more than 100 years. And, they must be aware of the fact that any given project only triggers a one-time CO_2_ saving effect. However, climate policy also demands from them a permanent periodical reduction in emissions. If they want to meet these requirements by means of the conservation strategy, they will have to frequently engage in REDD activities. This is substantially different from the avoidance of industrial emissions, which are based on permanently reducing emissions over a longer time period by means of a single new technology.

Investors require legal certainty with regard to their commitment. It is a fact that the destruction of forests is essentially a consequence of insufficiently defined and controlled land use rights. Forest areas whose ownership is unclear and which are illegally deforested cannot be subject to acquisitions. In order to integrate the endangered areas into the conservation policy, the necessary legal bases and control conditions must first be created.^6^ The most important driving forces behind the destruction of tropical rainforest areas are agriculture, cattle breeding and logging conducted by large businesses, small-holder farming as well as firewood procurement and public infrastructure projects. At least 50% of rainforest destruction is due to agriculture and cattle farming. It is important to negotiate with those groups. Even in places where the government is neither the owner nor administrator, it will often be involved in negotiations because it is jointly responsible for creating new living conditions for the previous users (small farmers, tenants, employees of large companies, etc.) and because, beyond the personal benefit of the owner, previous uses are of public interest (foreign exchange, licensing and tax revenues, etc.).

Forest conservation is considered attractive by industrialised countries and their investors because this type of emission avoidance is seen as particularly inexpensive compared to limiting emissions domestically, according to the general opinion. However, in the context of a concrete conservation policy system, the respective requirements regarding forest projects need to be taken into account. For the investors, this situation is not at all easy:

Investors must look for eligible suppliers and forest areas and must be convinced that the users are the entitled parties and their rights are sufficiently protected. The search is made difficult by the fact that at the present time, there are no organised markets for conservation projects.

The compensation for the previous owners waiving their rights of use is usually calculated in terms of opportunity costs. Thus, it is assumed that previous owners are integrated into the market economy and can seamlessly switch their source of income. The evaluation is carried out according to the discounted forgone yields from foregoing clearings and loggings.^7^ This approach is feasible for large companies in the agricultural and timber industries. Such companies, however, will not at all be satisfied with the capitalised values of the waivers of foregoing yields, but will demand a higher price.

Forests damaged by small farmers and local communities are a different matter.^8^ In order to achieve equivalent yields to the previous free use of the forests, they must incur costs, which are substantially higher than the previous yields. They are dependent upon modern methods of agriculture (e.g. using more efficient seeds, fertilisers and plant protection agents as well as access to loans and agricultural services), alternative fuels, the development of markets for products from sustainable cultivation (e.g. fruit, oils, resins) and employment opportunities in cities. For small farmers, all of this is unaffordable. Furthermore, these groups of people do not merely want to be compensated, but want to improve their situation, as the US model explicitly demands: local village communities and concerned individuals must be involved as stakeholders in the planning and execution of conservation projects and participate in the gains from the emission credits and allowances in a fair manner. If purchase decisions are made over the heads of village communities and their means of subsistence is taken away from them, they are left with no choice but to resort to other areas. In order to be able to deal with all the consequences of waiving their right of use, the small farmers need the government’s support. For the rainforest country, the question thus arises as to which costs of the infrastructural and the social consequences it will have to bear itself and which costs ought to be borne by the investors.

Project operators must deal with costs arising from the obligation to permanently sustain the forest area and protect it against destructive forces. They must consent to permanently conserving the forest stock in accordance with the generally accepted practices of sustainable forestry including the protection of original ecosystems and indigenous populations as well as holding off foreign biological influences. On the other hand, they might receive yields from sustainable forestry.

Investors must be prepared for discounting of the emission reductions as emission rights, without knowing the extent of it. This reduces the sales revenue from the credits and allowances and increases the investment risk. The uncertainty might be dispelled if a fixed standardised discount rate was determined for a given partner country; this, however, would devalue the macro concept of the baseline policy itself.

To summarise: the investors’ costs might be substantially higher than the opportunity costs for a project. Any references in current specialist literature indicating very low costs are of little help to investors interested in a specific project in a particular rainforest with a concrete conservation policy system. Rainforest conservation brings with it much higher risks than industrial prevention techniques (uncertainties in the authorisation process, an extensive long-term planning horizon, one-time climate effects of forest projects and an uncertain state of negotiations: who will be the investor’s partner and who will have what say during negotiations? Who will bear the social and infrastructural costs?).

## The rainforest country as investor of REDD projects

According to another approach put forward by rainforest countries, they would decide themselves whether to maintain forest projects. Private investors would not be part of the plan.^9^ As far as the basic design of this centralistic approach is concerned, it is not unlike the decentralised model discussed previously. A national baseline for deforestation emissions would be set and compared to the actual emissions of 1 year. If the actual emissions were to fall below that quantity, the difference would be remunerated at a price per tonne of CO_2_. An agreement would be made for the price to be paid out in cash or as emission allowances for the industrialised country. The payment would be made as soon as the service had been rendered, i.e., when the macro-balance was definite. The emission rights could come as credits or allowances. This approach allows for an innovation: if the actual emissions were to exceed the baseline during a particular period—due to special economic circumstances, a lack of enforcement or exogenous influences— excess emissions would be carried forward to the subsequent periods and settled against emission reductions.

This system leads to simplifications due to the omission of private investors. The evaluation and authorisation of single projects would be dropped. This way, the rainforest country would then receive more room for the design of adaptation measures. It would no longer be necessary to ensure a secure legal situation and to conduct the respective reform as well as controls necessary for creating legal certainty for private investors. However, a new problem would simultaneously arise. It would become increasingly difficult to negotiate a binding baseline between the contractual countries. In this system, the government of the rainforest country would make decisions both about the level of emission limits and the amount of additional forest activities. The higher the baseline value, the more supplementary services it could account for and request to have financed by the industrialised country. Furthermore, the government would be the recipient of remunerations from the emission rights in such a way that it could freely dispose of the revenue. These circumstances would promote the tendency to set an excessive baseline. It is difficult for the industrialised country to uncover manipulations in this area. In the decentralised system, however, the two areas of decision making would be separated.

If a reasonable baseline exists, there is increased financial pressure on the rainforest country to comply with and take the necessary measures for limiting the forest, for a lack of enforcement tends to manifest itself in lower public revenues. The government itself directly becomes the injured party. The same applies to cases in which conserved forest stocks are destroyed later on and the criterion of permanency is thereby breached. In the decentralised system, however, the private investors are not capable of exerting pressure on policy execution. Fulfilling the carry-forward rule also tends to have a positive effect on compliance: if an infringement of the baseline could occur within a current period, the respective emission excesses would lower the revenues, which could be achieved later, thus creating an incentive to put more effort into complying with the baseline. Such a macro penalty rule would not be applicable within the system of the decentralised US model. There, the political concept assumes a priori that actual emissions would regularly fall below the baseline.

The decentralised model turns negative when corruption poses a problem in the politics of the rainforest country. Because revenues from conservation programmes go directly to the government, they are easily accessed by corrupt politicians and civil servants. Funds that were intended for measures associated with the conservation of rainforests (legal reform, controls, measurements), for the compensation of the local populations concerned and for the promotion of the general economic development, would be diverted.

## How to proceed?

The global climate impact of a forest conservation programme depends to a great extent on a binding reference path for tolerable emissions from deforestation, which has been agreed upon and adhered to by the rainforest country by means of a forest law aimed at limiting deforestation. To this end, the national baseline approach with an appropriate contribution by the rainforest country coupled with a decentralised system with private investors seems the most viable option. Since additional burdens are imposed on the rainforest countries to some extent, a compromise could consist of agreeing on a moderate deforestation path, which is harmonised with the benefits from the forest projects. A reliable continuous measurement of the national forest stock is indispensible.

Combining both programmes (offset credits and emission allowances) is particularly attractive because all participants, and especially the industrialised country, benefit from it. The industrialised country can expand its climate conservation programme without any additional costs to a certain degree.

The conservation strategy holds risks for all parties involved. The industrialised country must reckon with releasing too many emission allowances and thereby missing its climate goal. Investors must thoroughly consider if and which forest projects will be beneficial to them compared with domestic avoidance techniques with regard to the potentially complex cost and risk conditions. Literature commonly suggests that avoidance costs for tropical rainforest projects in developing countries are very low, thus simplifying the matter too much. For the rainforest country, there is uncertainty as to whether it will be in the position to bear the costs and solve the socioeconomic conflicts which come with a baseline policy, including a reform of forest use rights, setting up an effective control system, carrying out continuous measuring of the forests’ carbon stocks and providing new sources of income for the previous forest users.

The question arises to what extent a rainforest country would need support with regard to its technical and institutional capacity. The US approach neglects this problem by demanding that agreements only be made with countries, which are already economically and institutionally capable of conducting an efficient baseline policy. A partner country offering these qualities would have proven in the past that it was able to solve conflicts of interest and that it had carried costs which no longer play a role after an agreement has been reached. Other types of costs would be new and additional, because special requirements of a forest conservation agreement must be met. There are likely only few countries capable of participating of their own accord in this programme anytime soon. One such candidate could be Brazil, which not only has the largest share in the destruction and damage of the global rainforest stock but also runs a satellite surveillance system for the rainforest and has introduced stricter controls and protection laws over recent years.

In the case of success, the conservation concept described previously could promote an advance in the international climate protection efforts in a twofold manner: first, by reducing current deforestation as an independent goal of the rainforest countries and second, by additional projects of private (and public) investors from abroad. The requirements and conditions would need to be laid down in an agreement so that all parties involved would benefit from it while national climate protection goals would more or less be reached. The developing countries, however, are not yet willing to legally commit to specific national emission limits; rather, they view their consent merely as political self-commitment. This causes uncertainties for the industrialised nations. But, unlike the general discussion about a new international climate agreement succeeding the Kyoto Protocol, rainforest programmes are faced with a special situation: the benefits from an agreement expected by a rainforest country disappear as soon as the country fails to stick to its commitments and the agreement is dissolved. This indirect penalty mechanism strengthens the trust of the industrialised countries in the contractual fidelity of the partner country.

## Footnotes

1 The following considerations essentially refer to the American Clean Energy and Security Act (ACESA) from 2009 which passed through the House of Representatives by a narrow majority, but failed in the Senate due to the Republicans' opposition.

2 Cf. Nobre (2010).

3 Cf. Larsen and Heilmayr (2009).

4 The US approach considers a subnational baseline to be an exception (for a federal state, a province or a region) if a partner country presently does not yet meet the requirements but is in a transition period to introduce the national baseline politics.

5 In The End of the Hinterland: Forest, Conflict and Climate Change (The Rights and Resources Initiative, Washington, D.C., 2009–2010) the following forms of ownership are distinguished: Administration by the government (A), Ownership of local communities and the indigenous population (B), Utilization determined by communities and the indigenous population (C), Ownership of individuals and companies. The following ownership structures were observed for 85% of the recorded rainforests in 2008: Africa 97.9% A; Asia 67.8% A, 26.5% B+C, 5.7% D; Latin America 36.1% A, 56.5 B+C, 7.3% D.

6 The main reasons for why little has been done so far to stop illegal deforestation are: Illegal deforestation relieves governments from problems of limited land availability due to a fast growing population; the importance of foreign currency revenue; the priority of economic growth interests; ignorance about the true extend of the deforestations due to infrequent forest stock taking; a lack of political enforcement against large-scale land owners and corruption.

7 According to the Stern Review (The Economics of Climate Change, http://www.hm-treasury.gov.uk, 2006, p 540) direct costs for agriculture, including logging, generally do not exceed $5 per tonne of CO_2_. For industrialised countries, however, market prices for emission rights are estimated about at least $15 per tonne of CO_2_—depending on the aspiration level for future climate conservation politics.

8 Cf. Gregersen (2010).

9 Cf. Federal Republic of Brazil, Brazilian Perspective on Reducing Emissions from Deforestation, 20 February 2007; Coalition of Rainforest Nations, Submission of Views: Reducing Emissions from Deforestation in Developing Countries: Approaches to Stimulate Action, San Josè, Costa Rica, January 30, 2007.
